# Thermal analysis of different shape nanoparticles on hyperthermia therapy on breast cancer in a porous medium: A fractional model

**DOI:** 10.1016/j.heliyon.2022.e10170

**Published:** 2022-08-11

**Authors:** Dolat Khan, Ata ur Rahman, Poom Kumam, Wiboonsak Watthayu, Kanokwan Sitthithakerngkiet, Ahmed M. Galal

**Affiliations:** a Fixed Point Research Laboratory, Fixed Point Theory and Applications Research Group, Center of Excellence in Theoretical and Computational Science (TaCS-CoE), Faculty of Science, King Mongkut's University of Technology Thonburi (KMUTT), 126 Pracha Uthit Rd., Bang Mod, Thung Khru, Bangkok, 10140, Thailand; b Center of Excellence in Theoretical and Computational Science (TaCS-CoE), Faculty of Science, King Mongkut's University of Technology Thonburi (KMUTT), 126 Pracha Uthit Rd., Bang Mod, Thung Khru, Bangkok, 10140, Thailand; c Department of Medical Research, China Medical University Hospital, China Medical University, Taichung, 40402, Taiwan; d Department of Mathematics, City University of Science & Information Technology, Peshawar, KPK, Pakistan; e Intelligent and Nonlinear Dynamic Innovations Research Center, Department of Mathematics, Faculty of Applied Science, King Mongkut's University of Technology North Bangkok (KMUTNB), 1518, Wongsawang, Bangsue, Bangkok, 10800, Thailand; f Department of Mechanical Engineering, College of Engineering in Wadi Alddawasir, Prince Sattam Bin Abdulaziz University, Saudi Arabia; g Production Engineering and Mechanical Design Department, Faculty of Engineering, Mansoura University, P. O. 35516, Mansoura, Egypt

**Keywords:** Nanofluid, Fractional derivative, Hypothermia therapy, Breast cancer, Transfer of bioheat, Biological tissues, Radiations

## Abstract

Cancer is clearly a major cause of disease and fatality around the world, yet little is known about how it starts and spreads. In this study, a model in mathematical form of breast cancer guided by a system of (ODE’S) ordinary differential equations is studied in depth to examine the thermal effects of various shape nanoparticles on breast cancer hyperthermia therapy in the existence of a porous media with fractional derivative connection, when utilizing microwave radiative heating. The unsteady state is determined precisely using the Laplace transform approach to crop a more decisive examination of temperature dissemination of blood temperature inside the breast tissues. Durbin's and Zakian's techniques are used to find Laplace inversion. Mild temperature hyperthermia is used in the treatment, which promotes cell death by increasing cell nervousness to radiation therapy and flow of blood in tumor. In the graphical findings, we can witness the distinct behavior of hyperthermia therapy on tumor cells by applying various metabolic heat generation rates across various time intervals to attain the optimal therapeutic temperature point. Particularly, we used graphs to visualize the behavior of different Nanoparticles with different shaped during hypothermia therapy. In comparison to other nanoparticles and shapes, it demonstrates that gold nanoparticles with a platelet shape are the best option for improving heat transmission. Which assess of heat transfer up to 16.412%.

## 1 Introduction

When breast cancer is diagnosed solely in the breast or adjacent lymph nodes and has not progressed to other regions of the body, it is called early breast cancer. Breast cancer is divided into subtypes based on whether hormone receptors, such as estrogen receptors, or other proteins play a role in the tumor's division and growth. In several countries around the globe, cancer is the leading cause of death. In women, breast cancer is the second most frequent cancer around the world [1]. A wide range of chemotherapeutic medication treatments are available for most forms of cancer, although in many clinical circumstances, no therapy regimen is demonstrably preferable. The benefits of taking various medications with antagonistic or synergistic actions have been established by Bonadonna *et al.* [2], Silvestrini *et al.* [3] and in two articles of Motwani *et al.* [4, 5]. Similarly, now a days many researchers are used nanoparticles for cancer treatments.

Nanomaterials are at the cutting edge of nanotechnology's ever-changing area. Because of their unique size-dependent features, these materials are exceptional and essential in a wide range of human activities. This brief study concludes by highlighting recent advances in the field of applied nanomaterials, with a focus on their use in biology and medicine, as well as their commercialization potential. The technology of Nano-particle deals with items as small as nanometers [6]. Nanotechnology is expected to advance on a variety of levels, such as materials, electronics, and systems. Nanomaterials are the most advanced at the moment, both in terms of commercial applications and scientific awareness. As the average diameter of a cell in a living mammal is ten millimeters. The cell fragments, are much smaller, measuring in the micrometer range. Proteins are even smaller, with a mean size of only 5 nm, which is equivalent to the smallest manmade nanoparticles. According to this simple size study, nanoparticles could be utilized as incredibly small probes to examine cellular machinery without creating too much damage [7]. Addressing biological activities at the nanoscale is a major driving force behind nanotechnology progress [8]. Furthermore, we are interested to study the use of different Nano-particles particularly in the various types of cancer therapies, and also their benefits.

Many investigators have used more targeted and relatively safer ways of hyperthermia to treat cancer since then, either alone or in combination with conventional therapy [9, 10, 11, 12]. Hyperthermia is a spike in the body tissues temperature, either locally or globally, in a medical environment. Other effects of hyperthermia include increased vascular perfusion and permeability, which improves tumors blood flow and oxygenation, and a change against anaerobic metabolism, which results in downturned the expenditure of oxygen and hiked tissue oxygenation, all of which lead to a transformed microenvironment in extracellular form [13, 14].

### 1.1 Nano particles for local hyperthermia

Tumors can be fed with intravenously delivered nanoparticles with a strong absorptivity for converting an external energy source to heat, maximizing energy conveyed in the tumor while reducing exposure to strong tissues. This technique of hyperthermia offers various potential advantages over hyperthermia achieved without nanoparticles, including global and focused hyperthermia. It is well known that nanoparticles delivered intravenously passively extravagate from the vasculature and aggregate preferentially in malignancies rather than normal tissues [15, 16]. To acquire even better tumor sensitivity, Nano-particles can be coated with focusing molecules that homing to cancer-associated and specific antigens of cancer [17, 18, 19, 20]. A smaller nanoparticle dose can have the same therapeutic effect while lowering the risk of nanoparticle induced damage. Conducted nanoparticles also produce little non-specific aggregation in the body.

### 1.2 Gold nano particles (GNPs) in cancer therapy

Hirsch et al. [21] were the first to show that (Gold Nano spheres) GNS mediated thermoablation is successful in a mouse tumor model. While early studies used direct injections of GNSs into subcutaneous tumors, later studies found that intravenously delivered GNSs combined in tumors as soon as 6 h after inoculation. Treatment of these mice with an 800 MW, NIR (Near Infrared) laser spouting at 808 nm, at 4 $W/cm^{2}$ for 3 min resulted in a substantial survival difference compared to non-radiated mice [22]. When gold Nano-particles are earnestly intended to tumors in mice, they improve survival compared to passively targeted nanoparticles [23, 24]. Another significant reason for including hyperthermia in tumor radiation therapy is to overcome the innate radio resistance of cancer stem tissues, which is regarded to be the fundamental cause of therapy collapse [25].

## 2 Superparamagnetic iron oxide nanoparticles (SPIONs) in cancer therapy

Prosperous hyperthermia requires the consignment of acceptable doses of SPIONs into tumors. Explicit injection into the lymphatic channels exhausting into the lymph nodes can transport them to probable metastatic cell nests in lymph nodes [26]. As proof-of-principle experiments, various researchers injected the SPIONs explicitly into the tumor. SPIONs can be put into hydrogels and organogels or injected into solution [27]. However, because of the evident problems in transferring SPION into practical use, this more intrusive approach of SPION delivery is not preferred. Intravenous delivery is the preferred method, however it is difficult to achieve successful nanoparticle concentration in tumors using this method. Loading SPIONs into cells is a unique method of delivery [28]. Tumor tropic neural progenitor tissues were weighted with SPIONs and injected into melanoma bearing mice, where the tissues entered the tumors. Tumor regression occurred after further exposure of autocrine motility factor (AMF). Heat can be used to treat cancer in two ways: ablative and sub-ablative. It can be applicable as a stand-alone cure to slow tumor hike [29] or as a radio sensitizer [25] when it is sub ablative. The AMF field can modulate the amount of heat generated by the tumor. However, elevating the voltage in the AMF generator, and thus the intensity of the AMF area, causes heat to be generated in tissues due to whirlpool current losses. This happens regardless of whether or not SPIONs are present, and it disturbs regular tissues in the AMF area. The amount to which the AMF field can be enhanced is limited by the increasing temperature in normal tissues. Radio-frequency disclosure of 25 kW for 20 min in mice, for example, elevated the temperature of tumor core up to 47 °C [30]. The toxicity of SPIONs to regular tissues in the nonexistence of AMF is another drawback of their use. Biocompatibility tests on human umbilical-vein endothelial tissues using citric acid-coated SPIONs and dextran [31] revealed the expected endocytosis absorption by the tissues, followed by cell death via hypothesized apoptotic pathways. The coated SPIONs were able to suppress the cells' migratory and invasion functions at concentrations as low as 0.1 mm While the coating/matrix of the SPIONs has a substantial influence on cytotoxicity, the findings highlight the importance of a full cytotoxicity assessment of any suggested therapy employing these nanoparticles.

### 2.1 Carbon nano-tubes (CNTs) in cancer therapy

The improved visual cross section of CNTs has been studied for photo thermal surgical removal of cancer parts, following in the pathway of gold Nano-rods SPIONs and (GNRs) [32]. Single wall carbon Nano-tubes (SWCNTs) have been used to demonstrate this in vitro [33, 34]. Tumor destruction has also been demonstrated in animal cancer models employing intra-tumoral injection of SWCNTs chased by 3 min of irradiation of NIR [35]. Multiple walls carbon Nano-tubes (MWCNTs), in addition to SWCNTs, have been shown to be successful in tumor ablation utilizing light NIR. However SWCNTs, MWCNTs can thermally ablate tumors with just a 30 s burst of light NIR at a modest laser power of 3 $W/cm^{2}$ [36]. Many other researchers are used carbon nanoparticles in their studies for other applications. As Acharya et al [37] investigate Carbon nanotube flow over a stretched surface in the presence of a magnetic field and Jia et al [38] reports the Brinkman type nanofluid model with carbon nanotubes. The readers can find some others investigation in [39, 40, 41].

However, in recent years, a body of plausible biological research has accumulated, and these data are now scientifically described. The goal of these mathematical models is to bring these disparate data sets together. Such mathematical models are essential for the development of new therapeutics and can also provide insights into the architecture of existing medications. The potential of a mathematical model to study the connection of noninfectious disease and provide valuable perceptions on contamination control and demeanor is well-known [42, 43]. Over the years, a mathematical model has been an important tool in recognition how illnesses work and in making decisions about medical cure approaches for breast cancer control in many nations [44]. The replication of mathematical models corresponded with tumor rise gesture and assessment about the cure, as well as irradiation alone, has recently been published [45]. Freedman and Nani [46] presented a mathematical model for cancer immunotherapy cure. In this post, they also examined the positives and cons. Many researchers looked into the control of surface temperature impact and circulatory systems of distribution of temperature on tissues during microwave heating in their research on hyperthermia therapy [47, 48]. Kritikos and colleagues [49] explored the temperature differential in a spherical area, which could lead to a hotspot in the center section of the brain. The Fourier transform approach was used to address the problem, demonstrating that the increase occurs in temperature for a large man's head is minimal. The mathematical formulation for microwave heating of half of the space took into account several similar temperature profiles, along with power law and exponential microphysical properties. The equation in differential form was solved numerically to get the results for the transient state issues, according to Hill and Income [50]. EI-dabe *et al.* [51], on the other hand, looked at the thermal state of living tissue when naked to microwaves. The authors used Maxwell equations with a bio thermal model to explore a one-dimensional multilayer model.

As we perceive that fractional calculus is more generalized variant of calculus. Fractional calculus considers all non-negative real numbers, unlike calculus of integer order, which target and based on integers. As a result, it's clear that using a fractional technique, a model's study, notably those linked to cancer, may be undertaken more thoroughly and the outcome seized more quickly. In this post, we take a more general and in-depth look at the problem using the fractional technique. With the above discussion in mind, we noticed that when employing a fractional technique, the impact of porous medium permeability and radiative heat flow of biological tissues were mostly disregarded in the literature. The fallout of the intensity of radiative heat flux and hyperthermia therapy on the tissues of breast in relation to porous medium permeability will be investigated using a fractional approach in this study. The work is being analyzed to build a more precise forecast of blood temperature distributions inside the breast tissues, and it is primarily for centrist temperature hyperthermia therapy of breast cancer in a severe location. The fallout of differences in heat source, porosity, blood thermal conductivity, thermal radiation, and blood perfusion on temperature distribution during fractional derivative microwave heating of hyperthermia therapy are an unresolved subject, according to the literature, which is the reason behind this study to perform.

### 2.2 Mathematical modeling and solution

During hyperthermia therapy, physical tissue that is initially at a constant T0=37C0 temperature is heated by an external heat source. The thermal flow process in living tissues combined with blood perfusion and circulation, tissues thermal conduction, and output of metabolic heat. Pennes [52] proposed a model to characterize the temperature of uniform arterial blood temperature in tissues, whereas temperature of venous blood is similar to the temperature of host tissues. The microwave heating equations in a one-dimensional multi-layer model are as follows in Eq. (1). [53].

(1) $${\left(\rho Cp\right)}_{nf}\frac{\partial \theta }{\partial t}=k_{nf}\frac{\partial^{2}\theta }{\partial y^{2}}+\omega_{b}{\left(\rho C\right)}_{nf}\left(\theta_{b}-\theta \right)+Q\left(\theta \right){\left|E\right|}^{2}$$

Moreover, porous media is preferable to [54, 55] since it demands less inputs than other bio heat transport models.

(2) $${\left(\rho Cp\right)}_{nf}\frac{\partial \theta }{\partial t}=k_{nf}\frac{\partial^{2}\theta }{\partial y^{2}}+\omega_{b}{\left(\rho C\right)}_{nf}\left(\theta_{b}-\theta \right)-\frac{\partial q}{\partial y}+\frac{\varepsilon }{k}+Q\left(\theta \right){\left|E\right|}^{2}$$

The following are the applied boundary conditions:

(3) θ(y,0)=37°C0,θ(0,t)=37°C0,θ(a,t)=45°C0,

The approximation of diffusion (Rosseland) is applied for radiation as in Eqs. (4), (5), and (6):

(4) $$q=\frac{-4\sigma }{3\sigma }\frac{\partial \theta^{4}}{\partial y}$$

(5) $$\theta^{4}≅4\theta_{b}^{3}\bar{\theta }-3\theta_{b}^{4}$$

(6) $$\frac{\partial q}{\partial y}=\frac{-16\sigma \theta_{b}^{3}}{3\delta }\frac{\partial^{2}\theta }{\partial y^{2}}$$

As a result, Eq. (2) gets:

(7) $${\left(\rho Cp\right)}_{nf}\frac{\partial \theta }{\partial t}=k_{nf}\left(1+\frac{4}{3}R\right)\frac{\partial^{2}\theta }{\partial y^{2}}+\omega_{b}{\left(\rho C\right)}_{nf}\left(\theta_{b}-\theta \right)+\frac{\varepsilon }{k}+Q_{m}\left(\theta -\theta_{0}\right)$$

The thermophysical correlations of nanofluids are as follows [56]

$$\;{\left(\rho c_{p}\right)}_{nf}=\left(1-\varphi \right){\left(\rho c_{p}\right)}_{b}+\varphi {\left(\rho c_{p}\right)}_{s}\;k_{nf}=k_{b}\left(\frac{k_{s}+\left(m-1\right)k_{b}-\varphi \left(m-1\right)\left(k_{b}-k_{s}\right)}{k_{s}+\left(m-1\right)k_{b}+\varphi \;\left(k_{b}-k_{s}\right)}\right),m=\frac{3}{\psi }\;\rho_{nf}=\left(1-\varphi \right)\rho_{b}+\varphi \rho_{s}$$

The following are the dimensionless variables [57].

(8) $$y=\frac{y*}{a},\;t=\frac{t*}{a^{2}},\;\vartheta =\frac{\left(\theta -\theta_{0}\right)}{\left(\theta_{b}-\theta_{0}\right)}$$

Eq. (9) is obtained by putting Eq. (8) in Eqs. (3) and (7),

(9) $$\frac{\partial \vartheta }{\partial t}=\frac{a_{2}}{a_{1}}\alpha_{1}\left(1+\frac{4}{3}R\right)\frac{\partial^{2}\vartheta }{\partial y^{2}}-\left(\gamma -\frac{\lambda }{a_{1}}\right)\vartheta +\left(\frac{\beta }{a_{1}}+\gamma \right)$$

The conditions are modified accordingly and listed below.

(10) ϑ(y,0)=37,ϑ(0,t)=37,ϑ(1,0)=45

where,

α1=kbρbCpb,β=a2εkbρbCpb(θb−θ0),γ=a2ω,bλ=a2QmρbCpbandR=−4σθb4kbδ

where $\alpha_{1},\;\beta \;,\;\gamma ,\;\lambda ,$ and R are Blood thermal conductivity, porous structure, Blood perfusion rate terms heat source and radiation parameters, respectively.

After applying the definition of time fractional Caputo derivative, Eq. (9) becomes:

(11) $${}^{C}D_{t}^{\alpha }\vartheta \left(y,t\right)=\frac{a_{2}}{a_{1}}\alpha_{1}\left(1+\frac{4}{3}R\right)\frac{\partial^{2}\vartheta }{\partial y^{2}}-\left(\gamma -\frac{\lambda }{a_{1}}\right)\vartheta +\left(\frac{\beta }{a_{1}}+\gamma \right)$$

By using Laplace transform on Eq. (11) and using Boundary condition in Eq. (10), we get

(12) $$q^{\alpha }\bar{\vartheta }\left(y,q\right)-q^{\alpha -1}\vartheta \left(y,0\right)=\frac{a_{2}}{a_{1}}\alpha_{1}\left(1+\frac{4}{3}R\right)\frac{\partial^{2}\bar{\vartheta }}{\partial y^{2}}-\left(\gamma -\frac{\lambda }{a_{1}}\right)\bar{\vartheta }+\left(\frac{\beta }{a_{1}}+\gamma \right)\frac{1}{q}$$

(13) $$\bar{\vartheta }\left(y,0\right)=\frac{37}{q},\;\bar{\vartheta }\left(0,q\right)=\frac{37}{q},\;\bar{\vartheta }\left(1,0\right)=\frac{45}{q}$$

Incorporating Eq. (13) in Eq. (12), we get the below form:

(14) $$\bar{\vartheta }\left(y,q\right)=A\;\cosh\left(y\sqrt{\frac{q^{\alpha }+a_{2}}{a_{1}}}\right)+B\;\sinh\left(y\sqrt{\frac{q^{\alpha }+a_{2}}{a_{1}}}\right)+\frac{37q^{\alpha }+a_{3}}{q\left(q^{\alpha }+a_{2}\right)}$$

$$\begin{array}{l} A=\frac{37}{q}-\frac{37q^{\alpha }+a_{3}}{q\left(q^{\alpha }+a_{2}\right)}\\ B=\frac{45}{q\;\sinh\left(\sqrt{\frac{q^{\alpha }+a_{2}}{a_{1}}}\right)}-\frac{37q^{\alpha }+a_{3}}{q\left(q^{\alpha }+a_{2}\right)}\left(\frac{\cosh\left(\sqrt{\frac{q^{\alpha }+a_{2}}{a_{1}}}\right)-1}{\sinh\left(\sqrt{\frac{q^{\alpha }+a_{2}}{a_{1}}}\right)}\right)-\frac{37}{q}\frac{\cosh\left(\sqrt{\frac{q^{\alpha }+a_{2}}{a_{1}}}\right)}{\sinh\left(\sqrt{\frac{q^{\alpha }+a_{2}}{a_{1}}}\right)} \end{array}$$

where

$$a_{1}=\frac{a_{2}}{a_{1}}\alpha_{1}\left(1+\frac{4}{3}R\right),\;a_{2}=\gamma -\frac{\lambda }{a_{1}},\;a_{3}=\frac{\beta }{a_{1}}+\gamma$$

The result of Eq. (14) is derived in the Laplace transform domain. The inverse Laplace transform of this equation is extremely difficult and complicated to manage, mainly in useful uses. As a result, numerical inversion is a more practical strategy to use. Table 1 validates the numerical solutions for Zakian's and Durbin's. Saqib et al. [60] apply the Durbin and Zakian inversion techniques in real-time.

**Table 1:** Thermo-physical properties [58, 59, 60].

	$\rho \left(Kgm^{-3}\right)$	$c_{p}\left(JKg^{-1}K^{-1}\right)$	$k\left(Wm^{-1}K^{-1}\right)$
Blood	1050	3617	0.52
Gold (Au)	19300	129	318
SWCNTs	2600	425	6600
MWCNTs	1600	796	3000
Iron oxide ($Fe_{3}O_{4}$)	5180	670	9.7

## 3 Results and discussion

**Figure 1:**
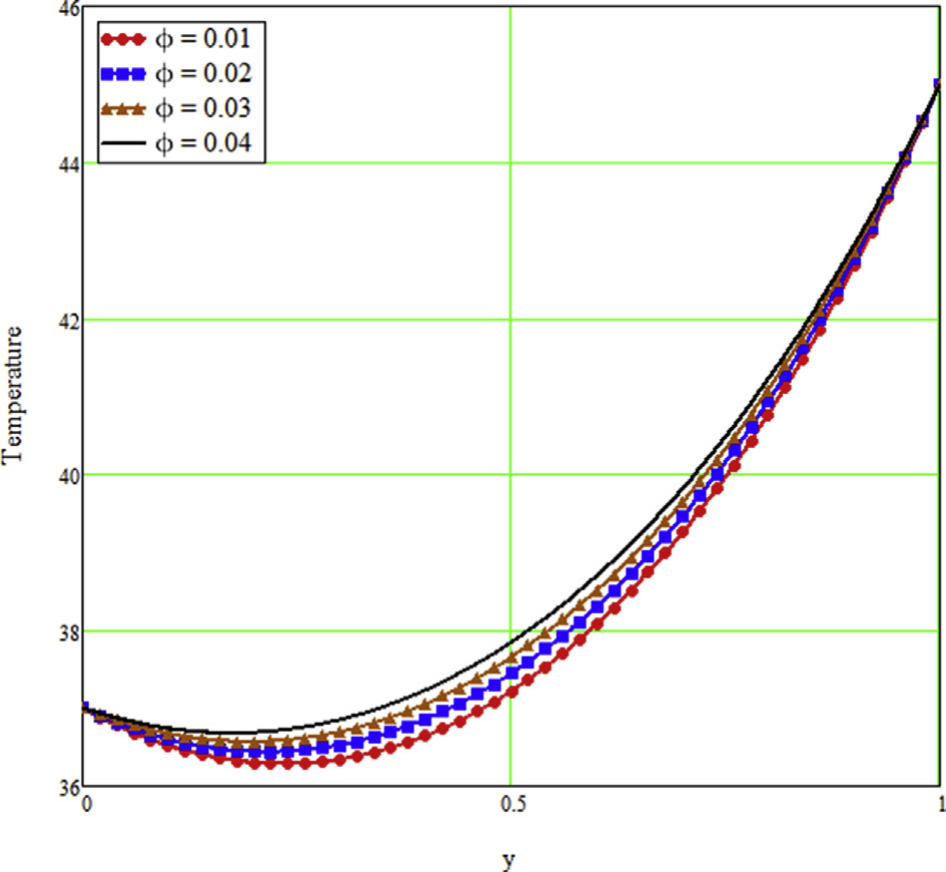
Effect of volume friction of nanoparticles on the field of temperature during hyperthermia therapy.

**Figure 2:**
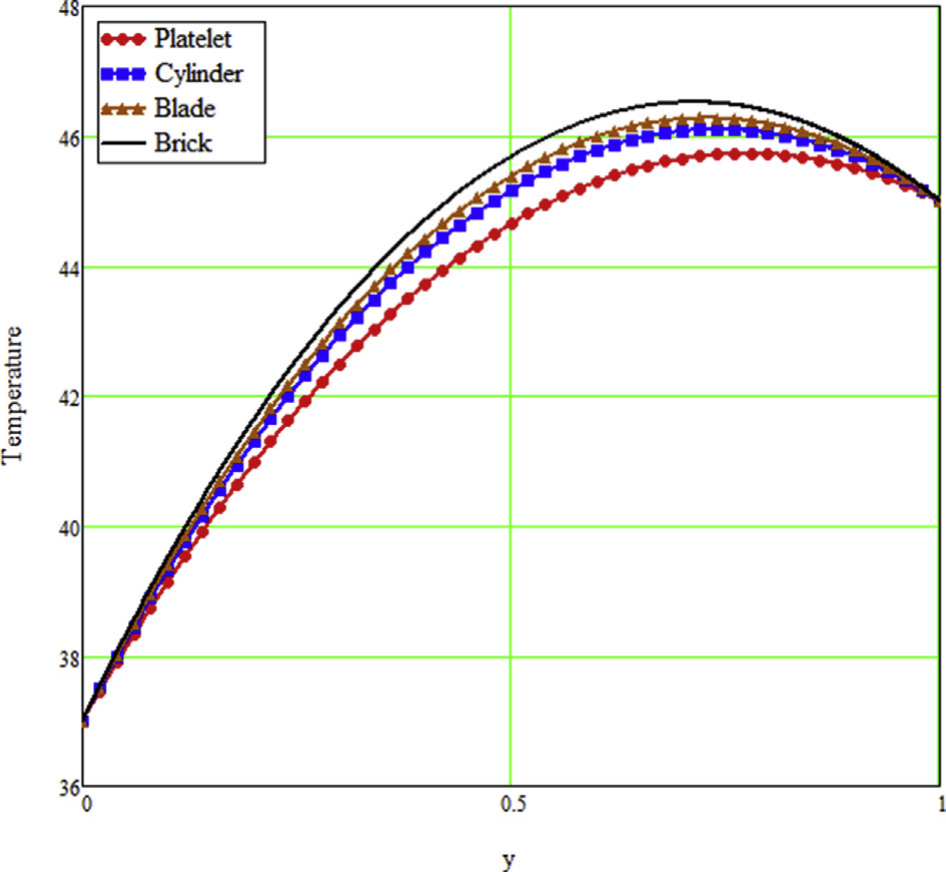
Effect of different shaped nanoparticles on the field of temperature during tumor hyperthermia therapy.

**Figure 3:**
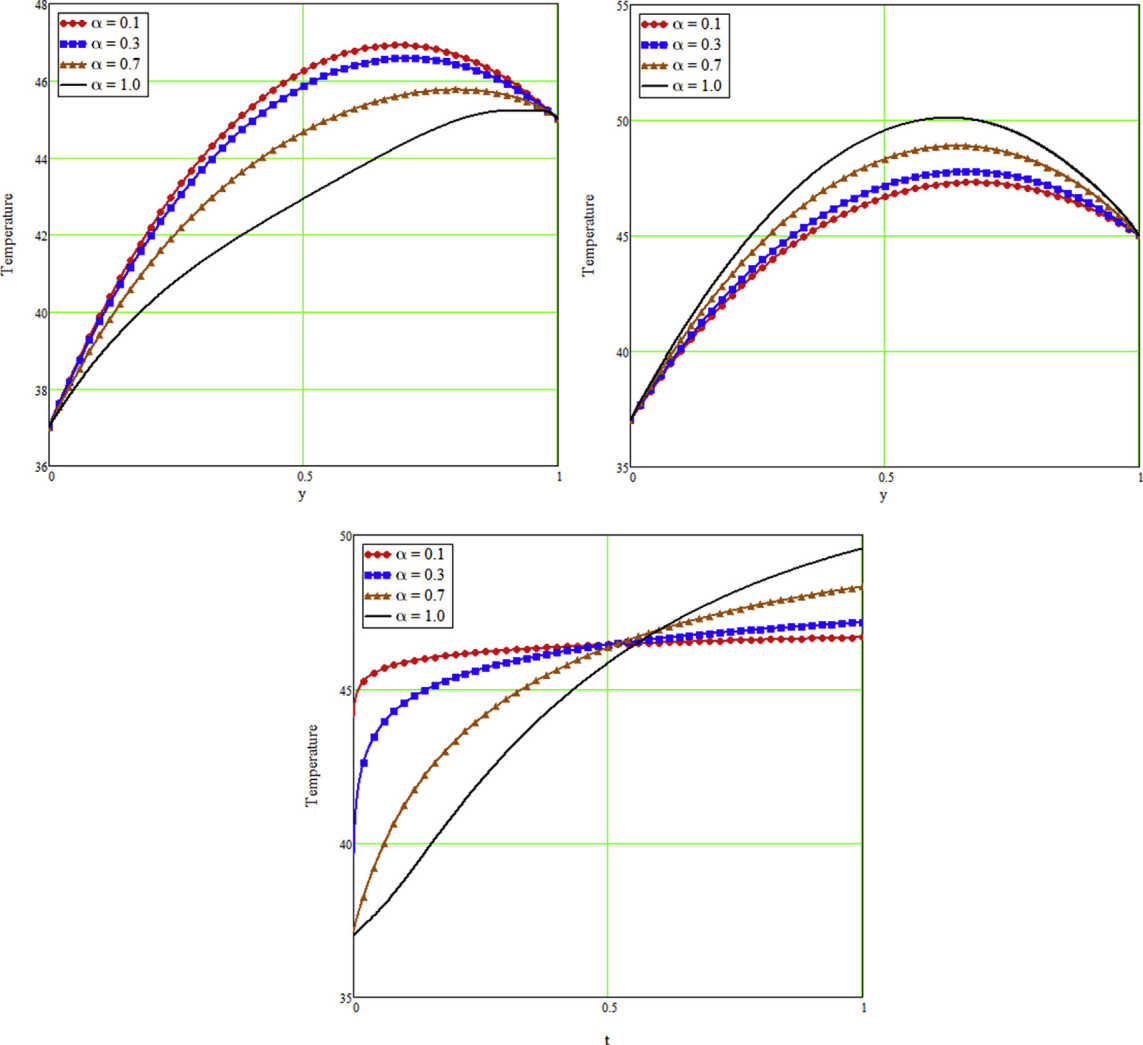
Fractional analysis on the field of temperature during tumor hyperthermia therapy.

**Figure 4:**
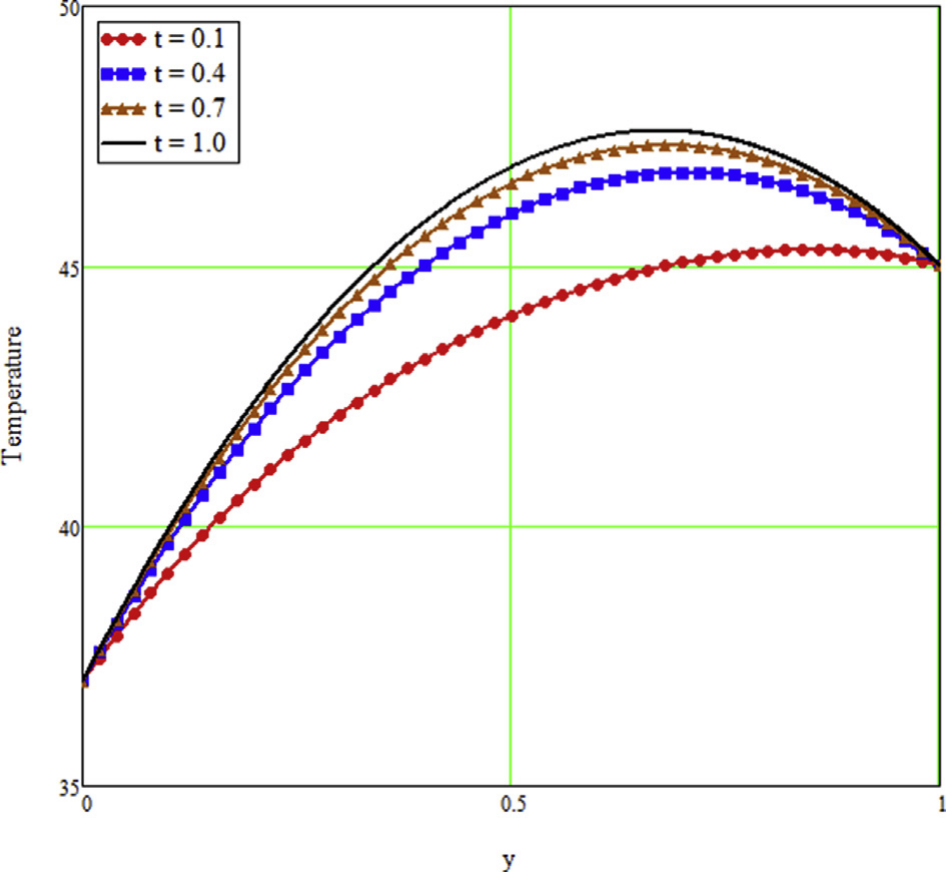
Effect of time on the field of temperature during hyperthermia therapy.

**Figure 5:**
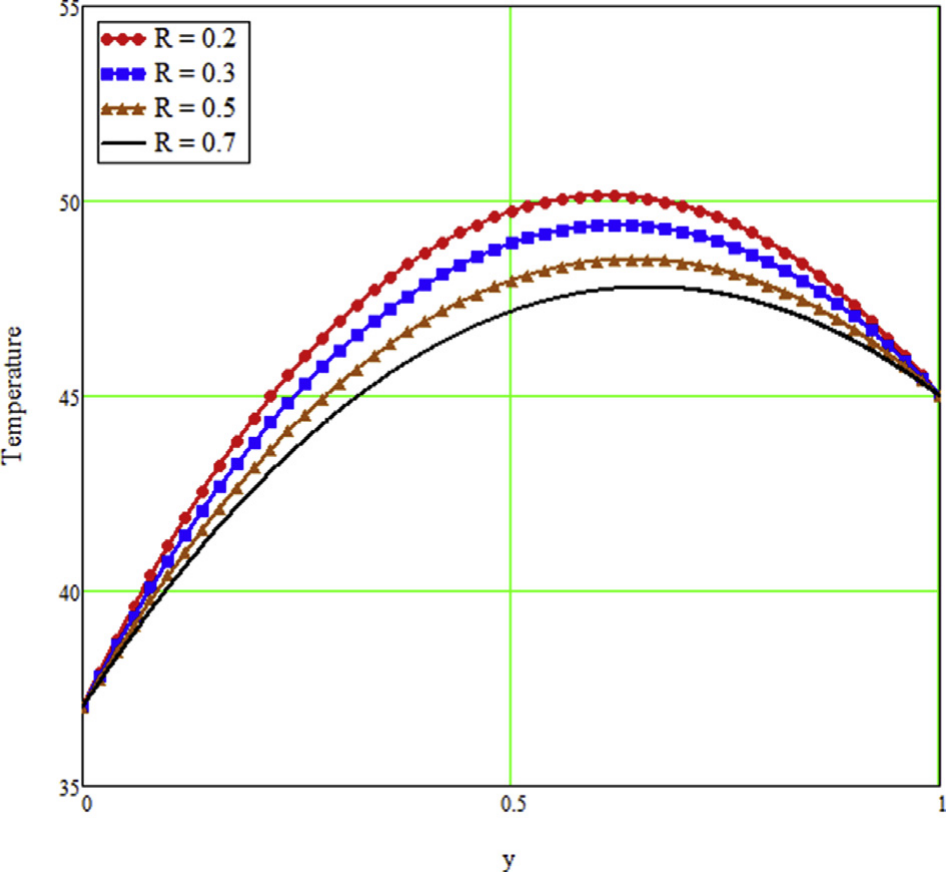
Effect of radiation parameters on the field of temperature field during hyperthermia therapy.

**Figure 6:**
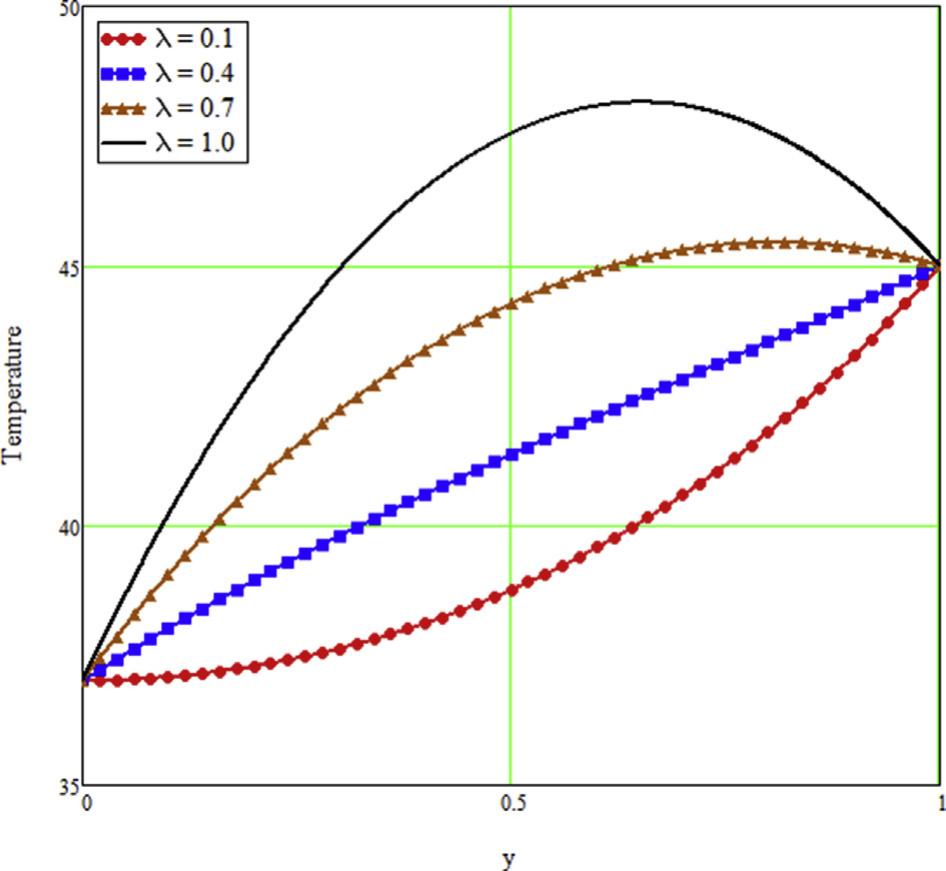
The effect of the heat source on the field of temperature during hyperthermia treatment.

**Figure 7:**
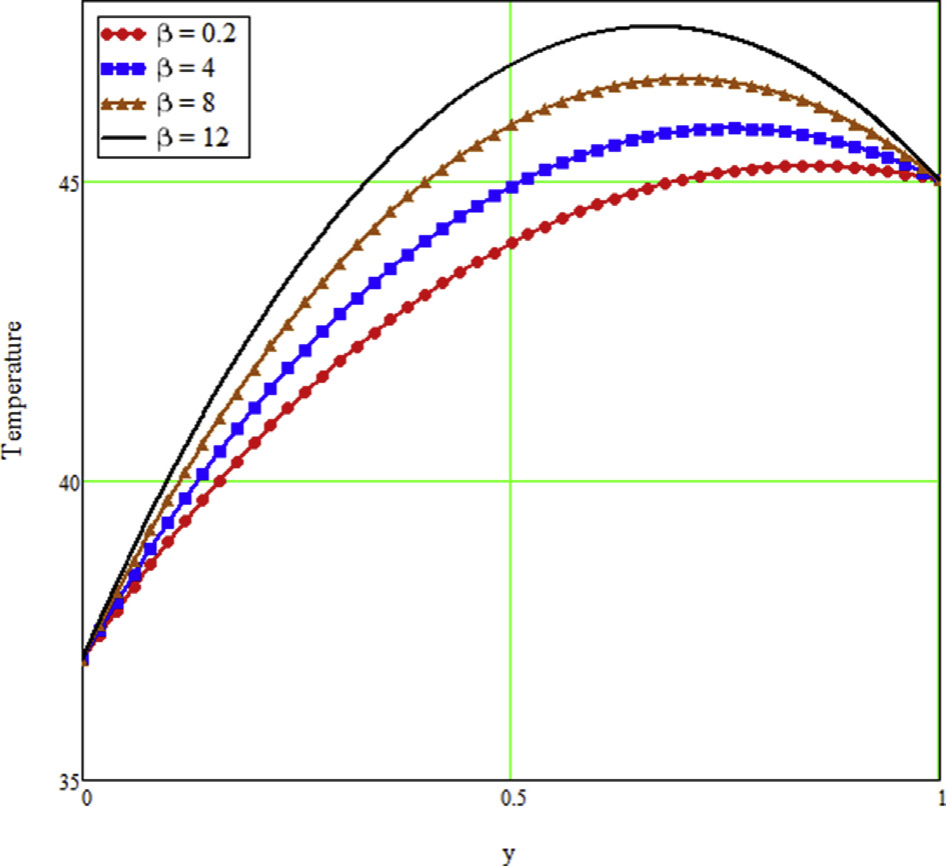
The effect of porous structure on the field of temperature during hyperthermia therapy.

**Figure 8:**
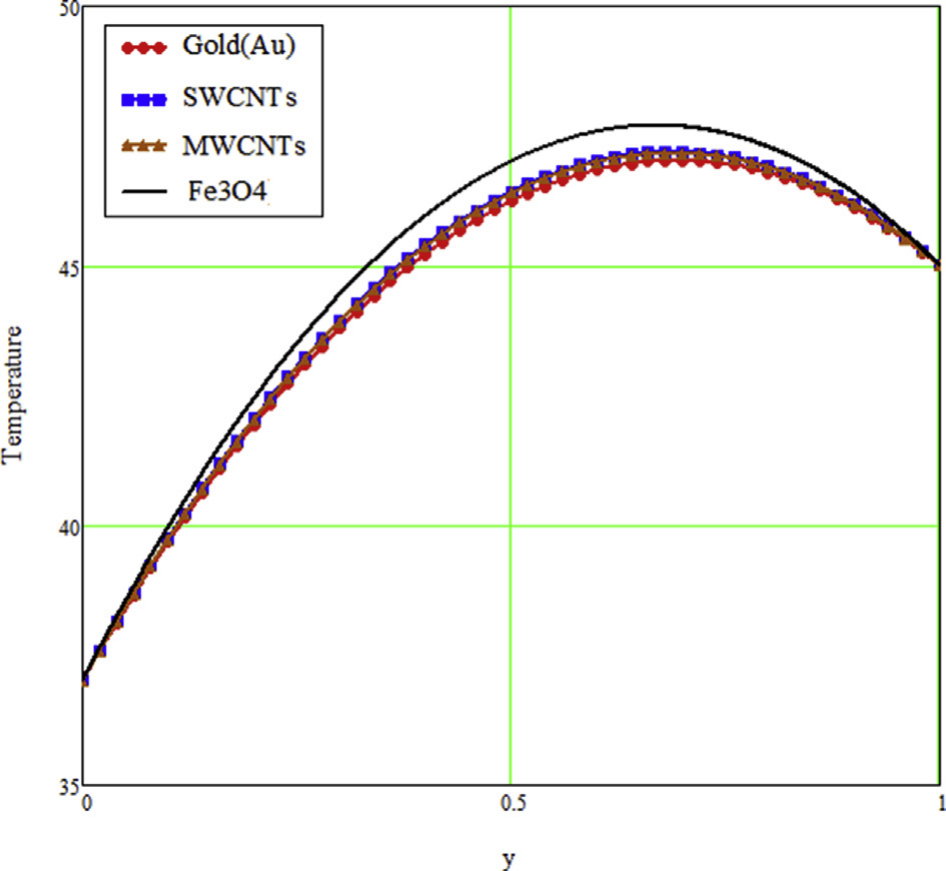
The profile of different nanoparticle based fluid on the field of temperature during hyperthermia therapy.

**Figure 9:**
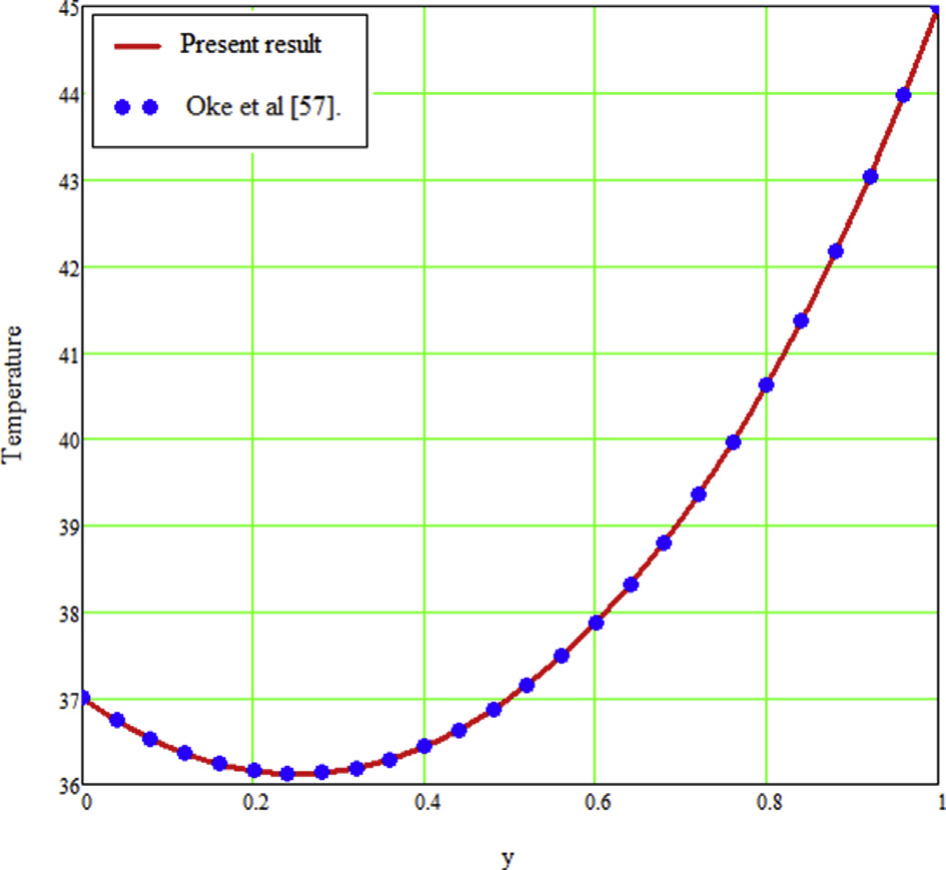
The comparison of the Present result with Oke et al. [57].

The ongoing study looks at a fractional technique of one-dimensional multilayer time reliant bio heat for detecting temperature in living biological tissue during microwave heating, such as breast tissues. Using the Mathcad-15 software, the results of the computational investigation were demonstrated in figures. Standard thermos-physical criteria for heat transmission in biological tissue necessitate a wide range of research. Boundary conditions and heat sources exist in the tissue as a result of metabolic heat production. Figure 1 interprets the relationship between the volume fraction of Nano-particles and temperature during hypothermia therapy, and it can be seen that as the volume fraction of Nano-particles increases, so does the temperature. From the temperature profile it can be easily noticed that the more is the concentration of nanoparticles in the fluid higher will be the viscous forces and consequently the fluid temperature increases as shown in figure. The main aim of Figure 1 to monitor the temperature against volume fraction of Nano-particles during hypothermia therapy. Hyperthermia, or the process of elevating the temperature of tumor-loaded tissue to 40–43 °C, is used in combination with other cancer treatments such radiotherapy and chemotherapy. The evolution of planning systems and other tools of modelling has substantially heightened the ability to manage power distributions in vivo recently. As a result of this improved understanding, multiantenna applicators and temperature monitoring systems have been developed. However, precise temperature management, both geographically and temporally, in deep body regions would further boost the potential for each temperature-dependent interaction used for clinical objectives. Figure 2 illustrates the performance of different shaped Nano-particles on temperature during hypothermia therapy, with platelet, cylinder, blade, and brick shaped Nano-particles exhibiting increasing behavior respectively. The volume of the various shaped Nano-particles is the physics behind the occurrence of an increase in temperature. The fraction increases as the volume of the particle increases, and the temperature rises as a result. Furthermore, the volume of a brick, blade, cylinder, and platelet is larger than the volume of the others, which explains why the fraction is also larger, and thus the temperature behaves correspondingly. Figure 3 presents the evolution of the fractional parameter. The discrepancy of the fractional parameter offers us collection of the different results, which clearly measures that the fractional phenomenon is utilized to generalize the result and identify the best data fitting for experimental inquiry. The memory effect is depicted in this graphic. Figure 4 shows the various temperature profile readings made during hypothermia therapy. During hypothermia therapy, the temperature rises with the passage of time, as shown in this graph. The use of therapeutic hypothermia necessitates continuous temperature monitoring of the core body. This is essential for attaining a precise goal temperature, avoiding overcooling, monitoring temperature changes during the maintenance phase, and guaranteeing a constant, regulated increase in temperature during the rewarming phase. The influence of the radiation parameter R on the temperature gradient is seen in Figure 5. Due to an increase in the effectiveness with which heat penetrate malignant cells as a result of radiotherapy, the temperature gradient grows as the value of R rises. The distribution of temperature after a discrepancy rise in the metabolic heat source is shown in Figure 6. The effective transmission of energy needed for chemical activities such as membrane pumps, muscular effort, glucose production from glucose, and protein construction from amino acids requires metabolic heat created in the tissue. Living tissue converts nearly all of the metallic energy captivated in these processes into heat. As the value of λ rises, the temperature profile rises, causing malignant cells to die. Figure 7 illustrates the demeanor of the porosity parameter β on the temperature profile. Temperature is clearly related to porosity, meaning that when the porosity parameter is increased, the temperature profile rises. The theory behind this phenomena is that, the term of porosity hike the flow of blood via tissues of porous, forcing blood cells to move quicker. Figure 8 illustrates the impact of different nanoparticles in a during hyperthermia therapy. It is clear that gold nanoparticle is the best choice to controls the temperature during hyperthermia therapy as compared to other three nanoparticles. It is because the density of the gold nanoparticle is greater then others. Figure 9 illustrate the comparison of the preset result with Oke et al. [57]. By putting fractional parameter α=1 and nanoparticle volume φ=0 the present results overlapped by the results obtained by Oke et al. [57], which validate the present results. Tables 2 and 3 highlighted the different nanoparticles and different shape nanoparticles impact on the appraise of heat transfer respectively. It shows that gold nanoparticle with platelet shaped is the best choice to augment the assess of heat transfer as compared to other nanoparticles and shapes. The gold nanoparticle augments the assess of heat transfer up to 16.412%, SWCNTs up to 13.869%, MWCNTs up to 13.649%, and $Fe_{3}O_{4}$ up to 8.247% while platelet shaped enhance the rate of heat transfer up to 16.412%, Cylinder shaped up to 12.006%, Blade shaped up to 10.378%, and Brick shaped up to 8.378%.

**Table 2:** Influence of different nanoparticles on Nusselt number.

φ	Gold		SWCNTs		MWCNTs		Fe3O4	
	Nu	%age	Nu	%age	Nu	%age	Nu	%age
0	20.88		20.88		20.88		20.88	
0.01	21.731	4.075	21.596	3.429	21.585	3.376	21.307	2.045
0.02	22.585	8.165	22.317	6.882	22.294	6.772	21.736	4.099
0.03	23.443	12.274	23.043	10.359	23.009	10.196	22.167	6.163
0.04	24.307	16.412	23.776	13.869	23.73	13.649	22.602	8.247

**Table 3:** Influence of shape effect on Nusselt number.

φ	Platelet		Cylinder		Blade		Brick	
	Nu	%age	Nu	%age	Nu	%age	Nu	%age
0	20.88		20.88		20.88		20.88	
0.01	21.731	4.075	21.499	2.964	21.415	2.562	21.311	2.064
0.02	22.585	8.165	22.123	5.953	21.954	5.143	21.745	4.142
0.03	23.443	12.274	22.752	8.965	22.498	7.749	22.185	6.25
0.04	24.307	16.412	23.387	12.006	23.047	10.378	22.629	8.376

## 4 Conclusion

This study focused on the mathematical model correspondence to breast cancer guided by a system of ODE's to examine the thermal effects of various shape nanoparticles on breast cancer hyperthermia therapy in the existence of a porous media with fractional derivative connection when utilizing radiative microwave heating. The unsteady state is determined precisely using the Laplace transform approach to crop a more decisive examination of the dissemination of blood temperature inside the breast tissues. Durbin's and Zakian's techniques are used to find Laplace inversion. Mathcad 15 were used to plot the graphs to know the physical parameters analysis on temperature profile. The main findings are summarized as follow:

• The volume fraction of nanoparticles is enhancing the rate of heat transfer during hyperthermia therapy.
• The different nanoparticles having ability to enhance the amount of heat transfer that is gold nanoparticles up to 16.412%, SWCNTs up to 13.869%, MWCNTs up to 13.649%, and $Fe_{3}O_{4}$ up to 8.247%.
• The shape of nanoparticles enhances the rate of heat transfer up to 16.412%,12.006%,10.378%, and 8.378% for Platelet, Cylinder, Blade and Brick shaped respectively.
• The fractional model makes the problem more general as compared to the ordinary on, which help the best curve fitting for the experimental data.
• Gold nanoparticle is having capability to control the temperature during hyperthermia therapy as compared to the other ones.

Finally, I would like to make some recommendations for further study to the readers, which are listed below.

• The following concept can be applied to more complex geometries like starching cylinder.
• One approach to investigating the same idea for several more generalized conditions.
• In addition, several novel fractional derivatives may be employed to more efficiently and realistic.

## Declarations

### Author contribution statement

Dolat Khan: Conceived and designed the experiments; Performed the experiments; Contributed reagents, materials, analysis tools or data; Wrote the paper.

Ata ur Rahman: Conceived and designed the experiments; Contributed reagents, materials, analysis tools or data; Wrote the paper.

Poom Kumam: Performed the experiments; Contributed reagents, materials, analysis tools or data.

Wiboonsak Watthayu: Conceived and designed the experiments; Analyzed and interpreted the data.

Kanokwan Sitthithakerngkiet: Analyzed and interpreted the data.

Ahmed M. Galal: Performed the experiments; Analyzed and interpreted the data.

### Funding statement

This research was supported by National Science, Research and Innovation Fund (NSRF) and King Mongkut’s University of Technology North Bangkok with Contract no. KMUTNB-FF-66-05.

### Data availability statement

No data was used for the research described in the article.

### Declaration of interest’s statement

The authors declare no conflict of interest.

### Additional information

No additional information is available for this paper.
